# Twin-Twin Transfusion Syndrome with and without Selective Fetal Growth Restriction Prior to Fetoscopic Laser Surgery: Short and Long-Term Outcome

**DOI:** 10.3390/jcm8070969

**Published:** 2019-07-03

**Authors:** Sophie G. Groene, Lisanne S. A. Tollenaar, Jeanine M. M. van Klink, Monique C. Haak, Frans J. C. M. Klumper, Johanna M. Middeldorp, Dick Oepkes, Femke Slaghekke, Enrico Lopriore

**Affiliations:** 1 Division of Neonatology, Department of Pediatrics, Leiden University Medical Center, 2333 ZA Leiden, The Netherlands; 2 Division of Fetal Medicine, Department of Obstetrics, Leiden University Medical Center, 2333 ZA Leiden, The Netherlands

**Keywords:** selective fetal growth restriction, twin-twin transfusion syndrome, survival, neurodevelopmental outcome

## Abstract

As twin-twin transfusion syndrome (TTTS) and selective fetal growth restriction (sFGR) are both prevalent complications of monochorionic (MC) twin pregnancies, its coexistence is not uncommon. The aim of this study is to evaluate the short and long-term outcome in TTTS with and without sFGR prior to fetoscopic laser coagulation. All TTTS cases treated with laser surgery at our center between 2001–2019 were retrospectively reviewed for the presence of sFGR, defined as an estimated fetal weight (EFW) <10th centile. We compared two groups: TTTS-only and TTTS + sFGR. Primary outcomes were perinatal survival and long-term severe neurodevelopmental impairment (NDI). Of the 527 pregnancies eligible for analysis, 40.8% (*n* = 215) were categorized as TTTS-only and 59.2% (*n* = 312) as TTTS + sFGR. Quintero stage at presentation was higher in the TTTS + sFGR group compared to the TTTS-only group (57% compared to 44% stage III). Separate analysis of donors showed significantly lower perinatal survival for donors in the TTTS + sFGR group (72% (224/311) compared to 81% (173/215), *p* = 0.027). Severe NDI at follow-up in long-term survivors in the TTTS-only and TTTS + sFGR group was present in 7% (13/198) and 9% (27/299), respectively (*p* = 0.385). Both sFGR (OR 1.5;95% CI 1.1–2.0, *p* = 0.013) and lower gestational age at laser (OR 1.1;95% CI 1.0–1.1, *p* = 0.001) were independently associated with decreased perinatal survival. Thus, sFGR prior to laser surgery is associated with a more severe initial presentation and decreased donor perinatal survival. The long-term outcome was not affected.

## 1. Introduction

Twin-twin transfusion syndrome (TTTS) is a disorder arising from inter-twin blood flow imbalances caused by vascular anastomoses on the surface of the shared placenta in monochorionic (MC) twins [1]. The donor transfers blood to the recipient, resulting in hypovolemia in the donor and hypervolemia in the recipient. Due to the decreased blood volume, the donor may present with growth restriction, next to the oliguria and oligohydramnios, while the recipient suffers from polyhydramnios [2]. If left untreated, TTTS has a high perinatal mortality rate [3]. Additionally, long-term outcomes show an increased risk of neurodevelopmental impairment (NDI) [4]. At present, fetoscopic laser surgery is the preferred treatment for TTTS, coagulating the placental vascular equator after selective coagulation of the anastomoses, called the Solomon technique [5,6].

Selective fetal growth restriction (sFGR) occurs in 10%–15% of MC pregnancies and is primarily due to unequal placental sharing [7]. sFGR is defined as an estimated fetal weight (EFW) <10th centile for the gestational age and/or a birth-weight discordance >25% [8]. Complications of sFGR include fetal deterioration, fetal demise, prematurity and neonatal morbidity or mortality [9]. NDI rates at follow-up appear to be high as well, with a disadvantage for the smaller twin [10,11,12].

As TTTS and sFGR are both prevalent complications of MC twin pregnancies, its coexistence is not uncommon. Approximately two-thirds of pregnancies complicated by TTTS present with coexistent sFGR, suggesting an underlying association between the pathogeneses of both disorders [13]. To understand this association better, placental analysis is necessary, with the quantification of the individual fetal territories. Moreover, coexistent sFGR prior to fetoscopic laser surgery might lead to a distinct course of the disease. More insight in the outcomes of TTTS with coexistent sFGR can thus result in a more specific prognostication, identifying risks and corresponding management options in an early stage. The aim of this study is to evaluate the impact of coexistent sFGR on short and long-term outcomes in MC twin pregnancies complicated by TTTS treated with fetoscopic laser coagulation.

## 2. Materials and Methods

In this retrospective study all consecutive TTTS cases treated with fetoscopic laser coagulation at our center between 2001 and 2019 were eligible for inclusion. Our center is the national referral center for fetal therapy in The Netherlands. From 2012 onwards, the Solomon technique was universally performed in our center, after conducting the Solomon trial from 2007 onwards. TTTS was diagnosed using the Eurofoetus criteria [14] and staged using the standard system [15]. All TTTS cases were reviewed for the presence of sFGR, defined as an EFW <10th centile prior to laser surgery. If so, the case was classified as ‘TTTS + sFGR’. If not, it was categorized as ‘TTTS-only’. The EFW before the laser had to be documented for both twins. We excluded MC triplet pregnancies, cases with concurrent twin anemia-polycythemia sequence (TAPS) [16], cases with incomplete laser surgery (either recurrent TTTS or post-laser TAPS) and cases in which a selective reduction or termination of pregnancy (TOP) was performed after a laser attempt.

The following maternal and neonatal baseline characteristics were collected from the medical records: Maternal age, gravidity, parity, gender, presence of umbilical artery (UA) Doppler abnormalities (defined as persistent or intermittent absent or reversed end-diastolic flow (A/REDF) prior to laser), Quintero staging, gestational age at birth, mode of delivery, birth weight, birth weight discordance (calculated as (birth weight large twin − birth weight smaller twin)/birth weight larger twin × 100%), proportion of pregnancies with a birth weight discordance >25%, proportion of neonates born small for gestational age (SGA) (defined as a birth weight <10th centile) and proportion of donor twins being the smaller twin at birth.

The number and type of placental anastomoses (arterio-venous (AV), veno-arterial (VA), arterio-arterial (AA) and veno-venous (VV)) was determined during the fetoscopic laser coagulation. After birth, routine placental injection was performed using colored dye to visualize vascular patterns at the placental surface [17]. The injected placenta was evaluated for residual anastomoses along the laser demarcation line, the placenta margin and in the membranes. Umbilical cord insertions were categorized into central, velamentous or marginal [18]. Fetal territories were differentiated by the margins of the twin-specific dyes and/or the laser demarcation line and expressed as a percentage of the total placental area. Placental share discordance was calculated as follows: (Placental share large twin − placental share small twin)/placental share large twin × 100%. 

Neurodevelopmental assessment was performed at two years of age using the Bayley Scales of Infant and Toddler Development second and third edition (Bayley-II and III) according to the routine care following fetal therapy in our center. Severe NDI was defined as cerebral palsy (CP) ≥grade II according to the Gross Motor Function Classification System (GMFCS) [19], impaired cognitive and/or motor development (IQ score <70 (−2SD)) or bilateral blindness and/or deafness (requiring amplification).

Primary outcomes were perinatal survival and long-term severe NDI. Secondary outcomes included fetal death (within 24 h of laser), neonatal mortality defined as death within 28 days after birth, disease-free survival defined as survival without severe NDI, and severe neonatal morbidity consisting of at least one of the following conditions: Respiratory distress syndrome (RDS) requiring mechanical ventilation or surfactant, patent ductus arteriosus requiring medical treatment or surgical closure, necrotizing enterocolitis ≥stage 2 [20] and severe cerebral injury (defined as cystic periventricular leukomalacia (PVL) ≥grade 2 [21], intraventricular hemorrhage (IVH) ≥grade 3 [22], ventricular dilatation >97th percentile [23], arterial or venous infarct, or porencephalic or parenchymal cysts). Outcomes were compared between TTTS-only and TTTS + sFGR. 

Statistical data were analyzed using the IBM statistics v23.0 (SPSS, Inc., an IBM company, Chicago, IL, USA). Data are presented as median with interquartile ranges, *n*/*N* (%) or *n* (%). A Mann-Whitney U test and a Chi-square test were used to analyze numerical data and categorical data, respectively. Data on survival and morbidity were analyzed using a Generalized Estimated Equation (GEE) when appropriate. A univariate linear regression model for identification of potential predictors of perinatal survival was performed. When antenatal baseline or placental characteristics differed between TTTS-only and TTTS + sFGR groups (*p* < 0.05), these were subsequently included in the univariate analysis along with characteristics previously described in literature. A separate analysis was performed for the TTTS + sFGR group to identify which cases are most at risk of perinatal death. When a significant association was found in the univariate analysis, the variable was included in the multivariate linear regression model. A *p*-value <0.05 was considered statistically significant for all analyses.

## 3. Results

**Figure 1:**
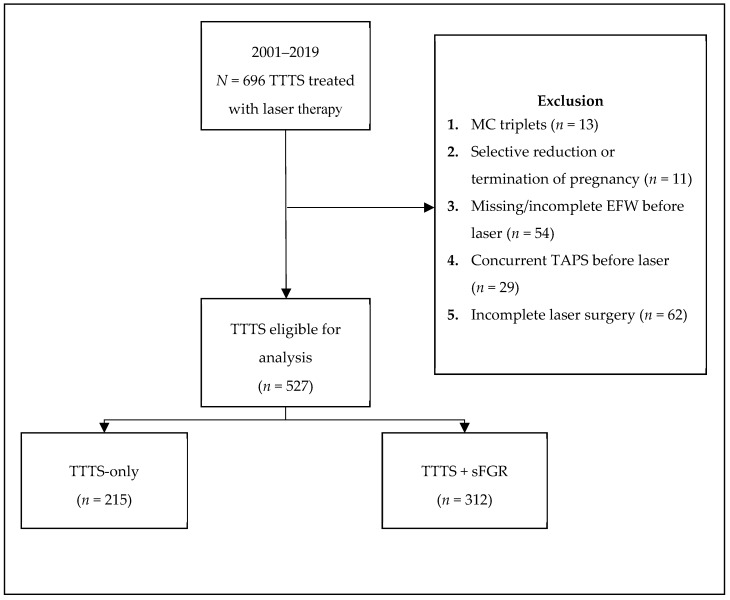
Flowchart of study inclusion. TTTS: Twin-twin transfusion syndrome, MC: Monochorionic, EFW: Estimated fetal weight, TAPS: Twin anemia-polycythemia sequence, sFGR: Selective fetal growth restriction.

Of the 696 twin pregnancies complicated by TTTS and treated with laser therapy, 54 had missing or incomplete records of EFW before laser coagulation (Figure 1). After exclusion according to the aforementioned criteria, 527 pregnancies were eligible for analysis, of which 40.8% (*n* = 215) were categorized as TTTS-only and 59.2% (*n* = 312) as TTTS + sFGR.

Eleven selective reductions/TOPs were performed for varied reasons, among which recurrent TTTS (*n* = 7), incomplete laser with severe growth restriction after laser (*n* = 1), fetal abnormalities (*n* = 1) and donor fetal demise with combined cerebral injury in the recipient (*n* = 2). All these cases were excluded from the analyses.

Baseline maternal and neonatal characteristics are summarized in Table 1. The TTTS + sFGR group had a significantly higher portion of females (53% (330/618) compared to 46% (196/424), *p* = 0.023). Of the 123 fetuses that presented with UA Doppler abnormalities prior to laser, 88% (108/123) were donors. In five cases, both donor and recipient simultaneously presented with similar UA Doppler abnormalities. Both persistent A/REDF (12% (73/592) compared to 4% (16/410)) and intermittent A/REDF (4% (24/592) compared to 2% (10/410)) were more frequently present in the TTTS + sFGR group (*p* < 0.0001). The distribution of Quintero stages differed significantly between groups. While most cases in the TTTS-only group had Quintero II stage at presentation (44%) the TTTS + sFGR groups presented predominantly with Quintero III stage (57%) (*p* < 0.0001). Gestational age at birth was 33.1 (29.3–35.9) weeks and 33.0 (29.7–35.7) weeks in the TTTS-only and TTTS + sFGR group, respectively (*p* = 0.780). In the TTTS + sFGR group, the birth weight of donor twins was lower and the birth weight discordance was higher compared to the TTTS-only group. The proportion of neonates born SGA was 21% (73/352) in the TTTS-only group and 49% (231/465) in the TTTS + sFGR group (*p* < 0.0001).

**Figure 2:**
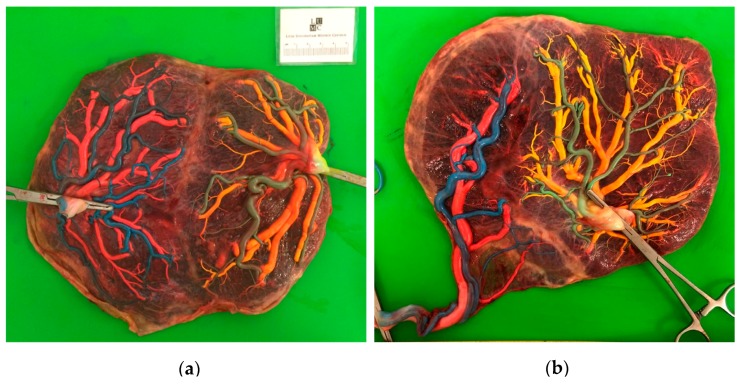
Pictures of injected placentas after birth, both treated with laser surgery, illustrating the placental share division of the individual fetuses: (**a**) Placenta of a TTTS-only case; (**b**) placenta of a TTTS + sFGR case.

A comparison of laser surgery outcomes and placental characteristics is presented in Table 2. Significant differences in placental angioarchitecture were detected between the two groups. Placentas in the TTTS + sFGR group more often had an AA anastomosis, velamentous cord insertions and unequal placental sharing (Figure 2).

**Figure 3:**
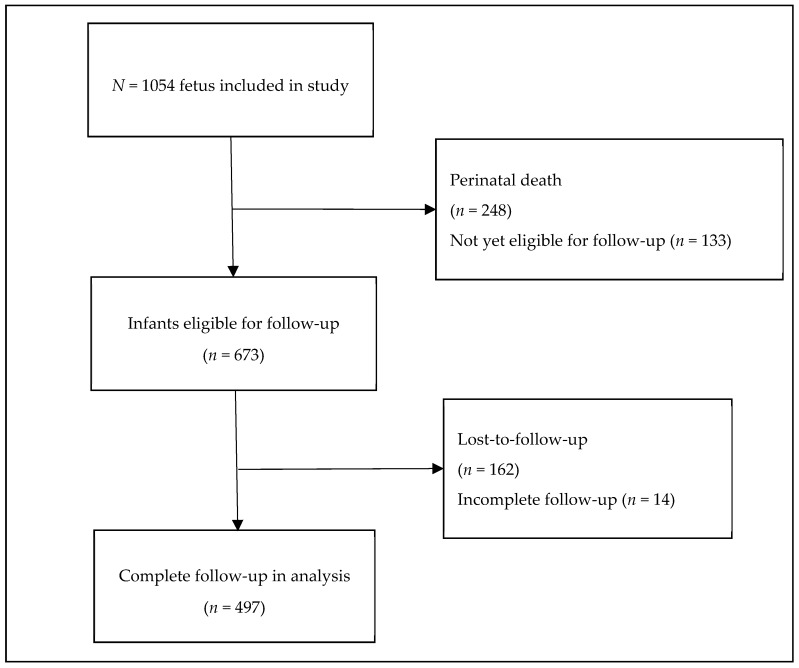
Flowchart of available long-term follow-up for analysis.

Short- and long-term outcomes are presented in Table 3. In our population, 673 infants were eligible for follow-up evaluation at two years, of which 162 (24.1%) were lost to follow-up, largely explained by lack of follow-up between 2006–2007 due to organizational issues (Figure 3). Baseline characteristics between infants with available follow-up and infants lost to follow-up did not differ, except for sex with 42% females lost to follow-up and 53% females with available follow-up (*p* = 0.010). Three neonates had no available information on neonatal mortality, and thus perinatal survival, as they were delivered elsewhere and we received no further information. Donor fetal death rate was 24% (76/312) in the TTTS + sFGR group and 17% (36/215) in the TTTS-only group (*p* = 0.036). Perinatal survival in the TTTS-only and TTTS + sFGR group was 79% (339/429) and 75% (464/622), respectively (*p* = 0.176). Separate analysis of donor and recipient showed a significantly lower perinatal survival for donors in the TTTS + sFGR group (72% (224/311) compared to 81% (173/215), *p* = 0.027). Severe NDI at follow-up in long-term survivors was present in 7% (13/198) and 9% (27/299) in the TTTS-only and TTTS + sFGR group, respectively (*p* = 0.385). Disease-free survival (including 248 deaths and 497 cases with complete follow-up) was found in 64% (185/288) of the TTTS-only group and 59% (269/457) of the TTTS + sFGR group (*p* = 0.159). Regarding other short-term outcomes, neonatal mortality for recipients was 6% (10/176) in the TTTS-only group as opposed to 1% (3/243) in the TTTS + sFGR group (*p* = 0.010).

Three factors appeared to be univariately associated with perinatal survival in the entire cohort: The presence of sFGR prior to laser surgery (OR 1.4;95% CI 1.1–1.8, *p* = 0.016), persistent A/REDF in the UA prior to laser (OR 1.9;95% CI 1.2–3.0, *p* = 0.006) and gestational age at laser (OR 1.1;95% CI 1.0–1.1, *p* = 0.003) (Table S1). After multivariate analysis, sFGR (OR 1.4;95% CI 1.1–1.9, *p* = 0.049) and gestational age at laser (OR 1.1;95% CI 1.0–1.1, *p* = 0.009) were independently associated with perinatal survival. The univariate linear regression analysis of potential predictors for perinatal survival in the TTTS + sFGR group was performed as well (Table 4). Persistent A/REDF in the UA prior to laser (OR 1.9;95% CI 1.1–3.1, *p* = 0.018) and gestational age at laser (OR 1.1;95% CI 1.0–1.2, *p* = 0.008) were univariately associated with perinatal survival. Both persistent A/REDF in the UA prior to laser (OR 1.7;95% CI 1.0–2.9, *p* = 0.049) and gestational age at laser (OR 1.1;95% CI 1.0–1.1, *p* = 0.023) were identified as independent risk factors for perinatal death.

## 4. Discussion

Our study shows that the donor perinatal survival is significantly decreased (72% compared to 81%) in TTTS cases with coexistent sFGR prior to laser surgery, mainly caused by an increased donor fetal death rate (24% compared to 17%). sFGR prior to laser surgery appeared to be an independent predictor of decreased perinatal survival. Within the TTTS + sFGR group, those with persistent A/REDF in the UA prior to laser (mainly donors) were especially at risk. Yet, the rate of severe NDI in long-term survivors at follow-up was not significantly different for TTTS-only and TTTS + sFGR cases. These findings imply that coexistent sFGR prior to laser surgery negatively influences perinatal survival, but does not affect short-term morbidities or long-term outcome. Moreover, we found that almost 60% of TTTS cases presented with coexistent sFGR, a percentage similar to what has been described by previous research [13]. As expected, TTTS pregnancies complicated by sFGR had a higher Quintero stage at presentation, as the Quintero III stage is per definition characterized by UA Doppler abnormalities.

In this study we did not only focus on the short and long-term outcomes, but also evaluated the placental differences between the three groups, since placental abnormalities are one of the most important substrates and causes of sFGR. As expected, individual placental shares were more unequally distributed in TTTS + sFGR cases. In line with typical placental characteristics of sFGR [24], both AA anastomoses and velamentous cord insertions were more frequently present in the TTTS + sFGR placenta’s. In isolated sFGR cases, AA anastomoses can allow for compensatory flow from the large twin to the smaller twin, resulting in partial reliance of the small twin on its co-twin for nutrients. Laser surgery in TTTS + sFGR cases results in occlusion of these anastomoses, possibly leading to further growth restriction or even fetal demise of the small twin (primarily the donor) as its already meager nutrient supply is cut even further. Moreover, laser surgery causes damage to the placenta, even though effort is made to minimalize this as much as possible. When a small twin loses even a small part of its already limited placental share, this can have detrimental consequences for its further growth and survival. These factors suggest that the increased risk of donor fetal death exists mainly due to iatrogenic damage to the placenta that twins with a small placental share cannot compensate for. Nevertheless, laser surgery is still the leading therapy for TTTS as it is live-saving for both twins, whether it might be more disadvantageous to the growth restricted twin or not. Our placental findings as well as the common coexistence of TTTS and sFGR illustrate the intricately related pathophysiology of both disorders originating from the unequal distribution and the angioarchitecture of the placenta.

Furthermore, the total proportion of neonates born SGA is 49% in the TTTS + sFGR group, implying that a proportion of growth restricted twins has experienced catch-up growth after laser. The prevalence of catch-up growth after laser surgery of growth restricted donor fetuses in TTTS cases has been described before [25]. However, this catch-up growth does not always occur. A distinction between two possible mechanisms of coexistent sFGR can be made. One results from the unequal placental sharing as described earlier, while the other results from the imbalanced flow where the donor relinquishes its nutrients to its co-twin. One can imagine that a donor twin with a relatively equally shared placenta but growth-restricted due to flow imbalances will benefit the most from the laser surgery. When the donor twin already has a small placental share, laser surgery might result in a bigger loss of placental share leading to further growth restriction. These two mechanisms possibly coexist together. Understanding them might lead to a better prediction of when catch-up growth after laser will or will not occur.

Our results concerning survival of TTTS cases with coexistent sFGR are similar to what has previously been reported [13]. Van Winden et al. found a markedly decreased 30-day survival rate of the donor twin in the TTTS + sFGR group (84% compared to 75%), very similar to our results. Patients in the TTTS + sFGR group were twice as likely to experience a donor fetal demise as compared to the TTTS-only group (20% compared to 10%). Although we observed a similar outcome, the effect of sFGR on donor fetal death rate was not as large as described by Van Winden et al. Lastly, they found that TTTS cases with donor twin growth restriction and UA Doppler abnormalities (Quintero III donor-involved) had the highest risk of 30-day non-survival. Similarly, we identified persistent A/REDF in the UA prior to laser as an independent risk factor for perinatal death in our TTTS + sFGR population. Thus, our results are in line with previous research, confirming the negative influence of coexistent sFGR on survival in TTTS cases.

Another study conducted by our own research group [26] identified an EFW discordance (OR 1.0;95% CI 1.0–1.1, *p* = 0.04), UA Doppler abnormalities (OR 3.0;95% CI 1.1–8.0, *p* = 0.01) and the presence of AA anastomoses (OR 5.6;95% CI 2.2–14.0, *p* = 0.03) as independent risk factors for single donor fetal demise after laser. This study population consisted of a small portion (*n* = 273) of our total population in the current study. We found a significantly increased donor fetal death rate for TTTS + sFGR cases and sFGR in the whole population and persistent A/REDF in the UA for the TTTS + sFGR cases as independent risk factors for perinatal death, supporting their results. However, our multivariate regression analysis did not identify an EFW discordance >25% or AA anastomoses prior to laser as risk factors for perinatal death. As we did not use an EFW discordance in our initial definition of sFGR and we did not solely analyze single fetal demise but also included double fetal demise and neonatal mortality, discrepancies between our results are not unimaginable. Hence, our studies should be regarded as separate entities describing specific distinctive outcomes. Importantly, the number of cases included in the current study is much larger, therefore increasing the statistical power and decreasing the likelihood of a Type II error.

Several limitations should be taken into account when interpreting our results. Due to the retrospective nature of the study some data were missing, possibly introducing bias into our results. Prospective research focused on TTTS and coexistent sFGR prior to laser surgery is necessary to provide further evidence of superior quality. Nevertheless, our results are reinforced by 1) the relatively large study population, 2) the elaborate placental analysis allowing for a better understanding of the pathophysiology, 3) the extensive documentation of neonatal morbidities and 4) the standardized long-term follow-up giving insight into the lasting effects of TTTS and coexistent sFGR on neurodevelopmental outcome. Combining these aspects, we think our study provides reliable insight in the short and long-term outcomes of TTTS + sFGR pregnancies.

In conclusion, this study shows that TTTS + sFGR prior to fetoscopic laser surgery is present in nearly 60% of TTTS cases and results from typical placental abnormalities (more unequal sharing, AA anastomoses and velamentous cord insertion). TTTS with coexistent sFGR is associated with a more severe initial presentation and a decreased donor perinatal survival, mainly due to an increased donor fetal death rate. sFGR was independently associated with decreased perinatal survival. Those with coexistent sFGR and persistent A/REDF in the UA prior to laser are especially at risk. The rate of long-term severe NDI does not seem to differ from TTTS cases without sFGR. Larger prospective studies are needed to confirm (or refute) our findings and provide more conclusive evidence. Yet, our extensive overview of short and long-term outcomes can aid clinicians in proper prognostication, risk factor identification and parent counseling in the future.

## Figures and Tables

**Table 1:** Baseline maternal and neonatal characteristics for TTTS-only and TTTS + sFGR.

Characteristics	TTTS-only (*n* = 430; 215 Pregnancies)	TTTS+sFGR (*n* = 624; 312 Pregnancies)	*p*-Value
Maternal age—years	32.0 (29.0–35.0)	31.0 (27.0–34.8)	0.334
Gravidity	2 (1–3)	2 (1–3)	0.587
Parity	1 (0–1)	1 (0–1)	0.298
Female	196/424 (46)	330/618 (53)	0.023
UA Doppler abnormalities			<0.0001
Persistent A/REDF	16/410 (4)	73/592 (12)	
Intermittent A/REDF	10/410 (2)	24/592 (4)	
Quintero stage			<0.0001
I	39 (18)	36 (12)	
II	94 (44)	87 (28)	
III	77 (36)	177 (57)	
IV	5 (2)	12 (4)	
Gestational age at birth—weeks	33.1 (29.3–35.9)	33.0 (29.7–35.7)	0.780
Caesarean	150 (36)	198 (33)	0.262
Birth weight—g			
Donor	1950 (1363–2415)	1640 (1093–2125)	<0.0001
Recipient	2005 (1540–2475)	1919 (1450–2303)	0.136
Birth weight discordance—%	8.1 (3.3–13.7)	15.6 (6.8–26.7)	<0.0001
Birth weight discordance >25%	10/161 (6)	61/205 (30)	<0.0001
Small for gestational age	73/352 (21)	231/465 (49)	<0.0001
Donor is smaller twin at birth	106/161 (66)	172/206 (84)	<0.0001

Data are median (interquartile range (IQR)), *n*/*N* (%) or *n* (%); TTTS: Twin-twin transfusion syndrome, sFGR: Selective fetal growth restriction, UA: Umbilical artery, A/REDF: Absent/reversed end-diastolic flow.

**Table 2:** Comparison of placental characteristics and laser surgery outcomes between TTTS-only and TTTS + sFGR cases.

Characteristics	TTTS-only (*n* = 430; 215 Pregnancies)	TTTS + sFGR (*n* = 624; 312 Pregnancies)	*p*-Value
Gestational age at laser—weeks	19.3 (17.4–22.0)	20.0 (17.9–22.1)	0.149
Total anastomoses at fetoscopy—*n*	6 (4–8)	6 (4–7)	0.412
AV anastomoses—*n*	4 (3–5)	3 (3–4)	0.176
VA anastomoses—*n*	2 (1–3)	2 (1–3)	0.822
Presence of AA anastomoses—*n*/*N* (%)	19/203 (9)	51/296 (17)	0.013
Presence of VV anastomoses—*n*/*N* (%)	19/203 (9)	28/296 (10)	0.970
Residual anastomoses at birth—*n*/*N* (%)	13/146 (9)	18/209 (9)	0.924
Cord insertion—*n*/*N* (%)			
Velamentous	61/330 (19)	116/440 (26)	0.007
Marginal	63/330 (19)	108/438 (25)	0.083
Placental share—%			
Donor	45.6 (41.2–50.7)	42.6 (33.6–50.0)	0.001
Recipient	54.4 (49.3–58.8)	57.3 (50.0–66.4)	0.002
Placental share discordance—%	22.1 (10.7–37.2)	33.6 (18.1–52.0)	<0.0001

Data are median (interquartile ranges (IQR)), *n*/*N* (%) or *n* (%). TTTS: Twin-twin transfusion syndrome, sFGR: Selective fetal growth restriction, AV: Arterio-venous; VA: Veno-arterial; AA: Arterio-arterial, VV: Veno-venous.

**Table 3:** Comparison of short- and long-term outcomes between TTTS-only and TTTS + sFGR cases.

	TTTS-only (*n* = 430; 215 Pregnancies)	TTTS + sFGR (*n* = 624; 312 Pregnancies)	*p*-Value
Donor fetal death	36 (17)	76 (24)	0.036
Within 24h of laser	9 (4)	27 (9)	0.404
Recipient fetal death	38 (18)	68 (22)	0.246
Within 24h of laser	13 (6)	31 (10)	0.377
Severe neonatal morbidity	66/287 (23)	86/418 (21)	0.593
Donor	32/144 (22)	36/204 (18)	0.289
Recipient	34/143 (24)	50/214 (23)	0.928
Respiratory distress syndrome	56/297 (19)	71/429 (17)	0.610
Donor	29/150 (19)	29/209 (14)	0.166
Recipient	27/147 (18)	42/220 (19)	0.862
Patent ductus arteriosus	14/295 (5)	8/429 (2)	0.100
Donor	6/149 (4)	2/209 (1)	0.053
Recipient	8/146 (6)	6/220 (3)	0.179
Necrotizing enterocolitis	9/292 (3)	11/429 (3)	0.770
Donor	4/147 (3)	4/209 (2)	0.613
Recipient	5/145 (3)	7/220 (3)	0.889
Severe cerebral injury	15/296 (5)	30/439 (7)	0.350
Donor	5/149 (3)	14/213 (7)	0.177
Recipient	10/147 (7)	16/226 (7)	0.918
Neonatal mortality	16/355 (5)	14/478 (3)	0.249
Donor	6/179 (3)	11/235 (5)	0.500
Recipient	10/176 (6)	3/243 (1)	0.010
Perinatal survival	339/429 (79)	464/622 (75)	0.176
Donor	173/215 (81)	224/311 (72)	0.027
Recipient	166/214 (78)	240/311 (77)	0.914
Severe NDI at 2-year follow-up	13/198 (7)	27/299 (9)	0.385
Donor	6/101 (6)	13/150 (9)	0.423
Recipient	7/97 (7)	14/149 (9)	0.550
Disease-free survival	185/288 (64)	269/457 (59)	0.176
Donor	95/143 (66)	135/237 (57)	0.067
Recipient	90/145 (62)	134/220 (61)	0.824

Data are *n*/*N* (%); TTTS: Twin-twin transfusion syndrome, sFGR: Selective fetal growth restriction, NDI: Neurodevelopmental impairment.

**Table 4:** Analysis of potential risk factors for perinatal death after laser surgery in the TTTS + sFGR group.

Characteristics	Perinatal Survival (*N* = 464)	Perinatal Death (*n* = 158)	Univariate Analysis OR (95% CI)	SE	*p*-Value	Multivariate Analysis OR (95% CI)	SE	*p*-Value
Severe sFGR (EFW <3rd centile)—*n*/*N* (%)	104/464 (22)	40/158 (25)	1.3 (0.9–1.9)	0.197	0.197			
EFW discordance >25%—*n*/*N* (%)	209/464 (45)	67/158 (42)	0.9 (0.6–1.4)	0.217	0.620			
UA Dopplers prior to laser—*n*/*N* (%)								
Persistent A/REDF	46/439 (11)	27/151 (18)	1.9 (1.1–3.1)	0.263	0.018	1.7 (1.0–2.9)	0.268	0.049
Intermittent A/REDF	18/439 (4)	6/151 (4)	1.0 (0.4–2.5)	0.481	0.946			
Female—*n*/*N* (%)	237/462 (51)	93/154 (60)	1.4 (0.9–2.2)	0.218	0.090			
Quintero stage—*n* (%)					0.847			
I	50 (11)	22 (14)	0.8 (0.2–2.8)	0.665				
II	132 (28)	42 (27)	1.0 (0.3–3.6)	0.623				
III	264 (57)	88 (56)	1.0 (0.3–3.2)	0.605				
IV	18 (4)	6 (4)	-	-				
Gestational age at laser—weeks	20.0 (18.0–22.4)	19.8 (17.0–21.6)	1.1 (1.0–1.2)	0.033	0.008	1.1 (1.0–1.1)	0.032	0.023
Presence of AA anastomoses—*n*/*N* (%)	36/222 (16)	15/73 (21)	1.3 (0.7–2.6)	0.342	0.397			
Velamentous cord insertion—*n*/*N* (%)	95/368 (26)	19/70 (27)	1.3 (0.8–2.0)	0.227	0.264			

Data are odds ratio (OR) (95% CI) and standard error (SE). OR: Odds ratio, CI: Confidence interval, SE: Standard error, sFGR: Selective fetal growth restriction, EFW: Estimated fetal weight, UA: Umbilical artery, A/REDF: Absent/reversed end-diastolic flow, AA: Arterio-arterial.

## Supplementary Materials

The following are available online at https://www.mdpi.com/2077-0383/8/7/969/s1, Table S1: Analysis of potential predictors of perinatal survival after laser surgery for TTTS.
