# Supplementary material for: Twin-Twin Transfusion Syndrome with and without Selective Fetal Growth Restriction Prior to Fetoscopic Laser Surgery: Short and Long-Term Outcome
Source: J Clin Med. 2019 Jul 3;8(7):969. doi: 10.3390/jcm8070969 (PMC6679158; doi:10.3390/jcm8070969)
Supplement: Supplementary file 1 [file jcm-08-00969-s001.pdf]

Journal of Clinical Medicine

Table 1. Analysis of potential risk factors for perinatal death after laser surgery for TTTS.

Characteristics	Perinatal survival (N=803)	Perinatal death (n=248)	Univariate analysis OR (95% CI)	SE	p-value	Multivariate analysis OR (95% CI)	SE	p-value
sFGR prior to laser – n/N (%)	246/803 (31)	96/248 (39)	1.4 (1.1-1.8)	0.324	**0.016**	1.4 (1.1-1.9)	0.152	**0.049**
Severe sFGR (EFW < 3rd centile) prior to laser – n/N (%)	104/803 (13)	40/248 (16)	1.4 (0.9-2.0)	0.191	0.097			
EFW discordance > 25% prior to laser – n/N (%)	232/803 (29)	70/248 (28)	1.0 (0.7-1.4)	0.188	0.853			
UA Dopplers prior to laser – n/N (%)								
Persistent A/REDF	57/760 (8)	32/239 (13)	1.9 (1.2-3.0)	0.235	**0.006**	1.5 (0.9-2.5)	0.251	0.093
Intermittent A/REDF	25/760 (3)	9/239 (4)	1.2 (0.5-2.5)	0.396	0.723			
Female – n/N (%)	391/799 (49)	135/240 (56)	1.3 (0.9-1.9)	0.290	0.097			
Quintero stage – n (%)								
I	117 (15)	33 (13)	1.7 (0.6-4.7)	0.522				
II	273 (34)	88 (36)	1.5 (0.6-3.8)	0.486	0.746			
III	390 (49)	116 (47)	1.6 (0.6-4.1)	0.479				
IV	23 (3)	11 (4)	-	-				
Gestational age at laser – weeks	19.9 (17.7-22.3)	19.4 (17.0-21.4)	1.1 (1.0-1.1)	0.026	**0.003**	1.1 (1.0-1.1)	0.025	**0.009**
Presence of AA anastomoses – n/N (%)	53/385 (14)	17/112 (15)	1.1 (0.6-2.0)	0.302	0.705			
Velamentous cord insertion – n/N (%)	151/659 (23)	23/108 (21)	1.1 (0.7-1.6)	0.195	0.757			

Data are odds ratio (OR) (95% CI) and standard error (SE).

OR: odds ratio, CI: confidence interval, SE: standard error, sFGR: selective fetal growth restriction, EFW: estimated fetal weight UA: umbilical artery, A/REDF: absent/reversed end-diastolic flow, AA: arterio-arterial
